# Morusflavone, a New Therapeutic Candidate for Prostate Cancer by CYP17A1 Inhibition: Exhibited by Molecular Docking and Dynamics Simulation

**DOI:** 10.3390/plants10091912

**Published:** 2021-09-14

**Authors:** Sayed Aliul Hasan Abdi, Amena Ali, Shabihul Fatma Sayed, Mohamed Jawed Ahsan, Abu Tahir, Wasim Ahmad, Shatrunajay Shukla, Abuzer Ali

**Affiliations:** 1 Department of Pharmacy, Al Baha University, Al Baha 1988, Saudi Arabia; sayedaliulhasan@gmail.com; 2 Department of Pharmaceutical Chemistry, College of Pharmacy, Taif University, P.O. Box 11099, Taif 21944, Saudi Arabia; amrathore@tu.edu.sa; 3 Department of Nursing, University College Farasan Campus, Jazan University, Jazan 54943, Saudi Arabia; Ssaid@jazanu.edu.sa; 4 Department of Pharmaceutical Chemistry, Maharishi Arvind College of Pharmacy, Ambabari Circle, Jaipur 302 039, Rajasthan, India; jawedpharma@gmail.com; 5 Department of Pharmacology, Raghukul College of Pharmacy, Bhopal 462 003, Madhya Pradesh, India; aliabutahir2009@gmail.com; 6 Department of Pharmacy, Mohammed Al-Mana College for Medical Sciences, Dammam 34222, Saudi Arabia; wasimahmadansari@yahoo.com; 7 Indian Pharmacopoeia Commission, Ministry of Health & Family Welfare, Government of India, Ghaziabad 201002, Uttar Pradesh, India; ankur.niperkolkata@gmail.com; 8 Department of Pharmacognosy, College of Pharmacy, Taif University, P.O. Box 11099, Taif 21944, Saudi Arabia

**Keywords:** Morusflavone, CYP17A1 inhibition, prostate cancer, molecular dynamics simulation

## Abstract

Morusflavone, a flavonoid from *Morus alba* L., was evaluated for its interactive ability and stability with CYP17A1, in comparison with abiraterone, which is a Food and Drug Administration (FDA)-approved CYP17A1 inhibitor. CYP17A1 inhibition is an important therapeutic target for prostate cancer. The CHAMM36 force field was used to perform molecular dynamics (MD) simulations in this study. The results show that Morusflavone has significant interactive ability and stability for CYP17A1, in comparison with abiraterone. The final interaction energies for the Morusflavone–CYP17A1 and abiraterone–CYP17A1 complexes were −246.252 KJ/mol and −207.86 KJ/mol, respectively. Since there are only limited therapeutic agents available, such as abiraterone, galeterone, and seviteronel, which are being developed for prostate cancer, information on any potent natural anticancer compounds, such as vinca alkaloids, for prostate cancer treatment is limited. The results of this study show that CYP17A1 inhibition by Morusflavone could be an important therapeutic target for prostate cancer. Further preclinical and clinical evaluations of the lead compound Morusflavone are required to evaluate whether it can serve as a potential inhibitor of CYP17A1, which will be a new hope for prostate cancer treatment.

## 1. Introduction

*Morus alba* L. (*Moraceae*) also known as a mulberry is a perennial, heterogeneous shrub. Mulberry grows in a variety of climates, from tropical to sub-arctic [1,2]. The *Morus alba* L., plant contains various phytochemicals like alkaloids, flavonoids, glycosides, terpenoids, steroids, volatile oils, tannins etc. The majority of the constituents isolated from *M. alba* have been reported for their biological properties [3,4,5,6,7,8]. However, the cytotoxicity of *M. alba* or its isolated compounds has been tested on a variety of human cancer cells, including lung carcinoma, breast carcinoma, hepatocarcinoma, colorectal and cervical cancers [7,8,9,10,11]. In addition, herbal constituents, such as Vinca alkaloids and S-allylcysteine, have been found to be useful for disease amelioration [10,12].

There are various treatment modalities for various types of cancers, and one such important treatment strategy for prostate cancer is CYP17A1 inhibition. The role of CYP17A1 and CYP17A1 inhibition in prostate cancer chemotherapy is an emerging therapeutic strategy for prostate cancer that functions by suppressing androgen production, as androgen is required for tumor growth. Huggins et al. established by clinical evidence that prostate morphogenesis is controlled by androgens and modulated by oestrogens, more than a half-century ago [13]. The androgen and its receptors are now recognized as key factors in the development of not only normal cancers but also prostate cancer [9,10].

Over the years, CYP17A1 inhibitors, such as abiraterone acetate, which is sold under the brand name Zytiga and is used to treat prostate cancer [14]. However, galeterone and seviteronel are being developed as treatments for prostate cancer. Ketoconazole is an older CYP17A1 inhibitor that is rarely used today, because it inhibits CYP17A1 competitively, its effectiveness is dependent on concentration [15]. In contrast, abiraterone acetate, once bound to CYP17A1, permanently (rather than competitively) disables it [14].

**Figure 1:**
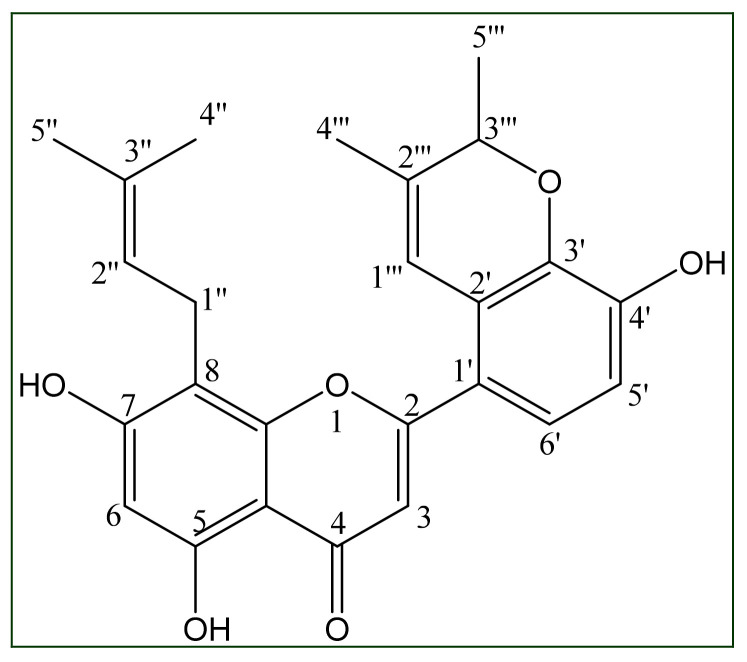
Morusflavone (5,7,4′-trihydroxy-8-(γ-methylallyl)-2′,3′-(2′′′,3′′′-dimethylpyr-1′′′-enyl) flavone).

Morusflavone is an isolated flavonoid from *M. alba* (*Moraceae*) stem bark (Figure 1). However, its stability and interactive efficacy to bind CYP17A1 has not been established. Therefore, in this study we have evaluated the stability and interactive efficacy of Morusflavone to inhibit the enzyme CYP17A1 in comparison with the Food and Drug Administration (FDA) approved inhibitor abiraterone by using molecular interaction and molecular dynamics simulations.

## 2. Results

### 2.1. Molecular Docking

**Figure 2:**
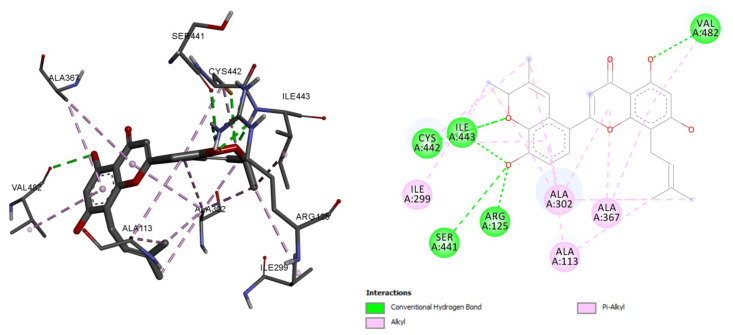
Morusflavone and its molecular interaction with CYP17A1.

For each ligand, molecular docking analysis was performed with Auto-Dock tools and 50 poses were generated as output. The binding energy scores of Morusflavone–CYP17A1 and abiraterone–CYP17A1 was found −9.53 and −9.22 Kcal/mol, respectively. The most interacting residues with Morusflavone and CYP17A1 were CYS, ILE, VAL, SER, ARG, and ALA. The residues associated with hydrogen bonds were CYS, ILE, VAL, SER, and ARG. However, the residues associated with alkyl and Pi-alkyl bonds were ILE and ALA, respectively (Figure 2).

**Figure 3:**
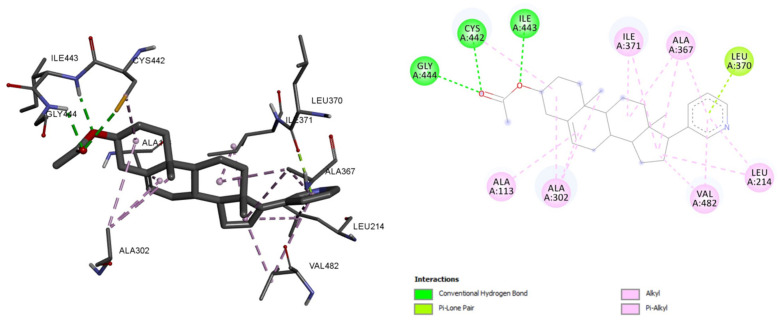
Abiraterone and its molecular interaction with CYP17A1.

The residues associated with the abiraterone–CYP17A1 molecular interaction were GLY, CYS, ILE, LEU, ALA, VAL, and LEU, whereas the residues which formed hydrogen bonds were GLY, CYS, and ILE. The LEU residue also formed Pi-lone pairs. However, ALA, VAL, LEU, and ILE were associated with Alkyl and Pi-alkyl bonds (Figure 3). The common residues between Morusflavone–CYP17A1 and abiraterone–CYP17A1 were ILE and CYS for hydrogen bonds; and ILE, and ALA for alkyl and Pi-alkyl bonds, respectively. The details of the molecular interaction analysis are given in Table 1.

### 2.2. Trajectory Analysis of the MD Simulation

The convergence of simulation was evaluated in terms of root–mean–square deviation (RMSD), root–mean–square fluctuation (RMSF), radius-of-gyration (RG) and number of intermolecular hydrogen bonds. In addition, the interaction energy and solvent-accessible-surface-area (SASA) were calculated for the ligand–substrate complex.

**Figure 4:**
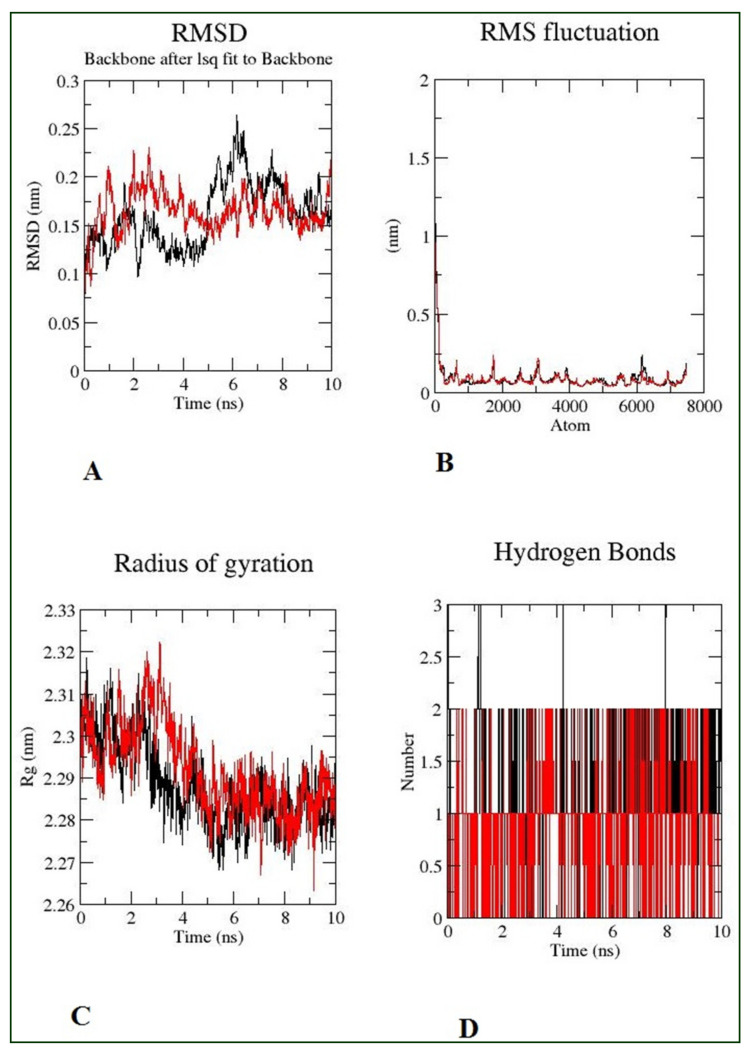
Pictorial presentation of the MD simulation. Morusflavone is represented by black and abiraterone by red. (**A**) RMSD for 10 ns; (**B**) The root–mean–square fluctuation for 10 ns; (**C**) The radius of gyration corresponds to 10 ns; (**D**) The number of hydrogen bonds formed between the ligand and CYP17A1.

The RMSD parameter analysis yielded detailed structural information about the conformational stability of each system. As a result, RMSD analyses were performed on both Morusflavone–CYP17A1 and abiraterone–CYP17A1. It was observed from analysis that Morusflavone–CYP17A1 and abiraterone–CYP17A1 were well equilibrated. Less than a ~0.05 nm (0.5 Å) deviation was observed in the CYP17A1 complex associated with Morusflavone, and abiraterone. After 6 ns, both complexes were superimposed, which indicated the similar stability of Morusflavone to CYP17A1 in comparison with abiraterone (Figure 4A). RMSF investigates the residue flexibility in the presence of a ligand; as depicted in Figure 4B, the RMSF value for the Morusflavone–CYP17A1 complex in comparison with abiraterone–CYP17A1 showed a similar fluctuation pattern that demonstrates restricted movement during the simulation. However, to analyze the overall compactness of the ligand–substrate complex, RG was estimated. As shown in Figure 4C, the RG was less, and the ligand and substrate were superimposed on each other. A lesser RG elucidates that the system was more compact, and vice versa.

**Figure 5:**
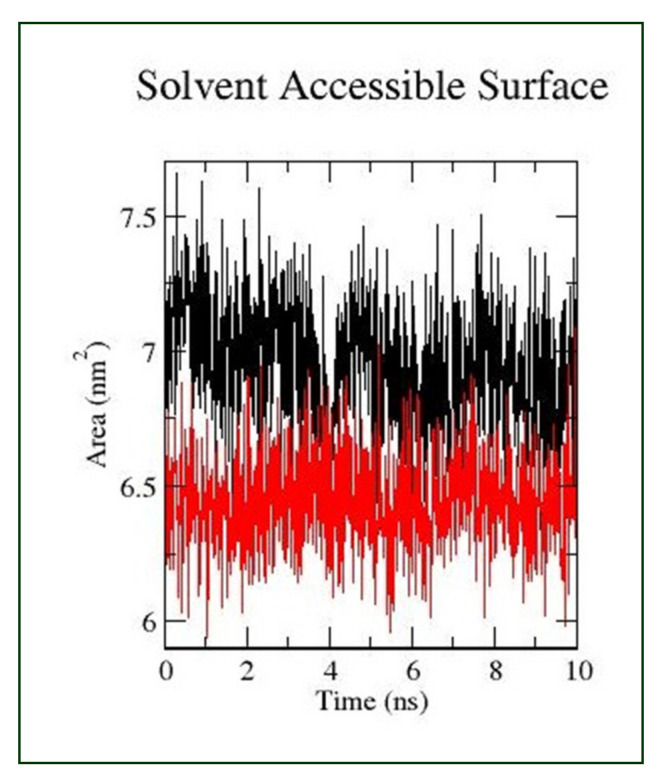
Pictorial presentation of SASAs. Morusflavone is represented by black and abiraterone by red.

Bond interactions are important for the overall stability of the protein structure. Intermolecular hydrogen bonds were investigated for the Morusflavone–CYP17A1 and abiraterone–CYP17A1 complexes (Figure 4D). The results illustrate that the enzyme–ligand complex of both ligands formed two intermolecular hydrogen bonds throughout the simulation. In addition, SASA was also calculated in this study, to discern the interaction of Morusflavone–CYP17A1 with solvents, in comparison with abiraterone–CYP17A1. The SASA also denotes conformational changes during the time of interactions. The results show that SASA of Morusflavone–CYP17A1 is similar to abiraterone–CYP17A1 (Figure 5). The interaction energy was also calculated in this study. These results show that Morusflavone–CYP17A1 has a higher potential to inhibit CYP17A1, with an interaction energy of −246.252 KJ/mol, in comparison with abiraterone–CYP17A1, which showed an interaction energy of −207.86 KJ/mol during MD simulation. Details of the stability analysis of the ligand–substrates are given in Table 2.

### 2.3. ADME and Toxicity Analysis

Absorption, distribution, metabolism, elimination, and toxicity (ADMET) exhibit important information about leading therapeutic candidates and the viability of a drug. The ADME and toxicity properties of Morusflavone were predicted by admetSAR 2.0. In many characteristics, Morusflavone showed significant values. The details of the ADME and toxicological profile are given in Table 3. As per the data in Table 3, Morusflavone appears to have safe toxicological profile.

## 3. Discussion

The most common neoplasia, among men in western countries, is prostate cancer (PC). As per epidemiological data, PC is a frequent cause of death among men in Greece [16,17]. However, several mechanisms have been postulated to change the phenotype of prostate cancer cells from hormone-sensitive to hormone-resistant [18]. These include either the specific upregulation of CYP17A1 or the overexpression of androgen receptors (AR) by cancer cells. The abrupt androgen amplification by gene upregulation may lead to altered AR activity [19]. In addition to abiraterone, other CYP17A1 inhibitors, such as orteronel, galeterone, VT-464, and CFG920 are being developed. However, there is a lack of potent natural CYP17A1 inhibitors.

In this study, Morusflavone, a flavonoid isolated from *M. alba*, and positively responded to the Shinoda test for flavonoids, was studied. Moreover, the various constituents of *M. alba* have been reported for their anticancer activity [20]. The in-silico screening of natural compounds has been extensively used in recent years [21]. We based this study on the interrelation between a natural compound, Morusflavone, derived from *M. alba,* and the enzyme CYP17A1. The binding energies for Morusflavone and abiraterone were −9.53 and −9.22 Kcal/mol, respectively. The common residues between Morusflavone and abiraterone were ILE and CYS, for hydrogen bonds, and ILE and ALA, for alkyl and Pi-alkyl bonds. These findings show that, in spite of the flavonoid structure of Morusflavone, it interacts with CYP17A1 in a pattern similar to abiraterone, which is a synthetic androstane steroid and a derivative of androstadienol. This reflects that Morusflavone has an inhibitory interactive behaviour with CYP17A1.

Comparative MD simulations were conducted to assess the effects of Morusflavone and abiraterone on the dynamical characteristics of the CYP17A1 protein, in either ligand-unbound proteins or ligand-bound proteins, to understand its inhibitory pattern in comparison with abiraterone. The RMSD, RMSF and RG of Morusflavone were similar to abiraterone. The overall dynamics of the Morusflavone–CYP17A1 complex caused a conservation in the dynamics of binding site regions similar to abiraterone-CYP17A1. The SASA of a biomolecule is the area of its surface that is accessible to a solvent and is an important property of proteins that influences their folding and stability [22,23]. The SASA findings revealed that the Morusflavone–CYP17A1 complex forms in aqueous solution driven by the CHARMM force field of the GROMACS-2019 software. Interestingly, the SASA of the Morusflavone–CYP17A1 complex was ~7.25 nm^2^, which was a little higher than the SASA of the abiraterone–CYP17A1 complex, which was ~6.6 nm^2^. It is reasonable to say that the behaviour of the flavonoid Morusflavone toward CYP17A1 is to find a stable binding interaction with a pattern similar to abiraterone–CYP17A1, leading to inhibition of CYP17A1, which justifies its ability as a lead therapeutic candidate. In addition, the complex’s interaction energy was also calculated by GROMACS-2019 software, which was also higher for Morusflavone, −246.252 KJ/mol in comparison with abiraterone, at −207.86 KJ/mol.

The ADME and toxicity parameters collectively assess the proper disposition of drug-like compounds in the body [24]. The data showed that Morusflavone has high GI absorption and has a predictive oral toxicity of category III (Category III includes compounds with LD50 values greater than 500 mg/kg but less than 5000 mg/kg). In addition, Morusflavone does not contravene Lipinski’s or Pfizer’s rules of five. Combining all the findings, it is possible to say that Morusflavone has the potential to form a stable interaction with the enzyme CYP17A1 that is similar to abiraterone, an FDA-approved inhibitor for prostate cancer. The findings of this study are encouraging and suggest that further preclinical and clinical study of Morusflavone is required, so that it can be clinically exploited against prostate cancer.

## 4. Materials and Methods

### 4.1. Extraction, Isolation and Structure Elucidation

Completely air-dried and coarsely powdered stem bark of *M. alba* (2 kg) was extracted with methanol, for 72 h, using a Soxhlet apparatus. Under low pressure, the extract was concentrated. For the preparation of slurry, dried extract (100 gm) was mixed into a small quantity of methanol and adsorbed onto silica gel. The slurry was dried before being subjected to column chromatography with silica gel (60–120 mesh). The elution of the silica gel in an open glass column with chloroform-methanol (19:1) resulted in pale yellow crystals of Morusflavone, which was re-crystallized from methanol. The structure of Morusflavone was established on the basis of UV, FT-IR, NMR, and MS data which was elucidated as 5,7,4′-trihydroxy-8-(γ-methylallyl)-2′,3′-(2′′′,3′′′-dimethylpyr-1′′′-enyl) flavone. The extraction, isolation and structural elucidation data of Morusflavone were published [3] (in Supplementary Materials, Data 1).

### 4.2. Molecular Docking

Morusflavone was docked with CYP17A1 (PDB ID-4NKY) using the Auto-Dock program (version 4.2) [25]. An FDA-approved CYP17A1 inhibitor (PUB Chem ID 9821849) was used as a comparator for molecular interaction and dynamics. Before beginning the analysis, the target’s structure was optimized by removing crystallographic water molecules and adding hydrogens to the protein in ideal geometry. The torsions were fixed, and the protein’s initial parameters, such as its Van der Waals and Kollman charges, were assigned and saved in PDBQT format. The grid was fixed and set to cover all parts of the active site’s enzymes. The grid-parameter files (GPF) and docking-parameter files (DPF) were created using the Auto-Dock tools, and the Lamarckian Genetic Algorithm was applied to generate potential protein–ligand binding conformations [26]. Finally, binding energies were calculated for 50 different poses. For the molecular dynamics study, a ligand–protein complex with a high binding energy was used [27].

### 4.3. Molecular Dynamics Simulation Analysis

Molecular dynamics was used in this study to examine the drug’s binding efficiency with the protein, overtime, at an atomic level. The RMSD, RMSF, RG, interaction-energy and SASA calculations were used in this study to examine the biophysical movement of the ligand-and-protein complex. In addition, intermolecular hydrogen bonds were also estimated in molecular dynamics (MD) simulation. The dynamic and structural changes in the enzyme–Morusflavone complex, in comparison with the abiraterone–enzyme complex, were subjected to MD simulation for a period of 10 ns by GROMACS-2019. The topological structure of the Morusflavone complex and the abiraterone–enzyme complex were parameterized by the CHARMM36 force field for all atoms [18]. Simple point charge (SPC) was used for enzyme–ligand solvation, and counter ions (Cl or Na) were applied for neutralization [28].

For the minimization of energy, Van der Waals contacts between the atoms were excluded. The equilibration was performed in two-phased NVT and NPT, which were constant number of particles, volume, temperature, and a constant number of particles, pressure, and temperature, respectively, for 10 ns. The system was ensemble at 300 K, and the linear constraint solver (LINCS) algorithm was applied to constrain the covalent bonds. MD simulation was estimated at 10 ns with Morusflavone and abiraterone, along with CYP17A1 [27].

### 4.4. ADME and Toxicity Analysis

The pharmacokinetic properties and toxicity of Morusflavone were evaluated by the admetSAR 2.0 (http://lmmd.ecust.edu.cn/admetsar2/, accessed on 15 July 2021) web-based server. Before undergoing a drug trial, it is critical to assess the harmful effects of chemical compounds. Toxicity testing is a critical step in the drug-development process [29].

## 5. Conclusions

The current study sought to identify the efficacy of Morusflavone, in comparison with abiraterone, in interacting stably with CYP17A1, which is a therapeutic target for prostate cancer. As per pharmacoinformatic data, Morusflavone has significant ADMET and physicochemical properties. The most obvious finding from the MD simulation results is that Morusflavone has stable interactions with CYP17A1 that are similar to abiraterone, which is evident from the SASA, interaction-energy, RMSD, RMSF and RG analyses. The current findings emphasize the significance and efficacy of Morusflavone to stably interact with CYP17A1, so that various preclinical and clinical studies on Morusflavone may be conducted by researchers.

## Figures and Tables

**Table 1:** Molecular interaction analyses with protein (4NKYCYP17A1).

Ligand	H-Bonds	H Bond Distance (Å)		Amino Acid Residues Involved in Hydrophobic Interactions	Docking Final Intermolecular Energy (ΔG) = vdW + Hbond + Desolv Energy (Kcal/mol)	Inhibition Constant (*Ki*)
		Between Hydrogen and Acceptor Atom	Between Donor and Acceptor Atom			
Morusflavone	ARG 125	3.01	3.53	PHE 114	−10.3	103.87 nM
	ILE 443	1.76	2.60	ALA 302		
	VAL 482	2.03	2.75	ILE 443		
				VAL 482		
Abiraterone	ILE 443	3.01	3.71	ILE 443	−10.07	174.43 nM
	GLY 444	2.31	2.85	ALA 113 302, 367		
	CYS 442	2.18	2.86	PHE 114		
				VAL 482		

**Table 2:** Stability analysis of ligand–substrate.

Enzyme Substrate Complex	RMSD (nm)	RMSF (nm)	RG (nm)	SASA (nm^2^)	Interaction Energy (KJ/mol)
Morusflavone–CYP17A1	0.05 ± 0.02	0.25 ± 0.01	2.29 ± 0.02	7.25 ± 0.01	–246.252
Abiraterone–CYP17A1	0.05 ± 0.02	0.25 ± 0.01	2.31 ± 0.02	6.6 ± 0.01	–207.86

**Table 3:** Important ADME and toxicological properties of Morusflavone.

S. No.	Parameters	Results
1	Lipinski violation	No
2	No. of H-bond acceptors	6
3	No. of H-bond donors	3
4	Molecular weight	420.45 g/mol
5	GI absorption	High
6	BBB permeation	No
7	CYP1A2 inhibitor	No
8	CYP2C19 inhibitor	No
9	CYP2C9 inhibitor	Yes
10	Log *Kp* (skin permeation)	−5.14 cm/s
11	Acute Oral Toxicity (c)	Category III
12	Eye irritation	No
13	Ames mutagenesis	Yes

## Data Availability

The authors confirm that the data supporting the study’s findings are included in the article and its Supplementary Information.

## Supplementary Materials

The following are available online at https://www.mdpi.com/article/10.3390/plants10091912/s1, Data 1: Characterization data of Morusflavone.
