# Supplementary material for: Morusflavone, a New Therapeutic Candidate for Prostate Cancer by CYP17A1 Inhibition: Exhibited by Molecular Docking and Dynamics Simulation
Source: Plants (Basel). 2021 Sep 14;10(9):1912. doi: 10.3390/plants10091912 (PMC8468843; doi:10.3390/plants10091912)
Supplement: Supplementary file 1 [file plants-10-01912-s001.zip › plants-1347370-supplementary.pdf]

Article

Morusflavone, a new therapeutic candidate for prostate cancer by CYP17A1 inhibition: Exhibited by molecular docking and dynamics simulation

Sayed Aliul Hasan Abdi [1], Amena Ali [2], Shabihul Fatma Sayed [3], Mohamed Jawed Ahsan [4], Abutahir [5], Wasim Ahmad [6], Shatrunajay Shukla [7], Abuzer Ali [8]*

[1] Department of Pharmacy, Al Baha University, Kingdom of Saudi Arabia;sayedaliulhasan@gmail.com
[2] Department of Pharmaceutical Chemistry, College of Pharmacy, Taif University, P.O. Box 11099, Taif 21944, Saudi Arabia; amrathore@tu.edu.sa
[3] Department of Nursing, University College Farasan Campus, Jazan University, Kingdom of Saudi Arabia;Ssaid@jazanu.edu.sa
[4] Department of Pharmaceutical Chemistry, Maharishi Arvind College of Pharmacy, AmbabariCircle,Jaipur 302 039, India; jawedpharma@gmail.com
[5] Department of Pharmacology, Raghukul College of Pharmacy, Bhopal, Madhya Pradesh 462 003, India; aliabutahir2009@gmail.com
[6] Department of Pharmacy, Mohammed Al-Mana College for Medical Sciences, Dammam 34222, Saudi Arabia; wasimahmadansari@yahoo.com
[7] Indian Pharmacopoeia Commission, Ministry of Health & Family Welfare, Government of India, Ghaziabad, Uttar Pradesh, India; ankur.niperkolkata@gmail.com
[8] Department of Pharmacognosy, College of Pharmacy, Taif University, P.O. Box 11099, Taif 21944, Saudi Arabia; abuali@tu.edu.sa

* Correspondence: abuali@tu.edu.sa (Abuzer Ali)

Supplementary electronic material

Morusflavone: UV (MeOH, λ_{max}, nm): 241, 264, 310 (log ε 4.1, 4.2, 3.7) [1]; IR (KBr, ν_{max}, cm^{-1}): 3399, 3210, 2965, 2913, 2855, 1680, 1653, 1623, 1554, 1445, 1417, 1361, 1291, 1237, 1165, 1132, 1054, 978, 836 [1]; ^{1}H NMR (DMSO-d_6, 300 MHz): δ ppm,6.45 (1H, brs, H-3), 6.28 (1H, brs, H-6), 7.07 (1H, d, *J* = 8.4 Hz, H-5′), 6.37 (1H, d, *J* = 8.4 Hz, H-6′), 5.13 (1H, brs, H-1‴), 3.26 (1H, m, H-3‴), 1.57 (3H, brs, Me-4‴), 1.39 (3H, d, *J* = 6.5 Hz, Me-5‴), 3.24 (1H, dd, *J* = 7.3, 6.6 Hz, H_2-1″a), 3.03 (1H, dd, *J* = 7.3, 6.3 Hz, H_2-1″b), 5.03 (1H, brt, *J* = 6.6 Hz, H-2″), 1.52 (3H, brs, Me-4″), 1.53 (3H, brs, Me-5″) [1]; ^{13}C NMR (DMSO-d_6, 75 MHz): δ ppm 160.83 (C-2), 102.76 (C-3), 181.61 (C-4), 160.03 (C-5), 97.68 (C-6), 161.41 (C-7), 106.53 (C-8), 158.79 (C-9), 105.30 (C-10), 130.55 (C-1′), 119.25 (C-2′), 154.87 (C-3′), 156.15 (C-4′), 111.29 (C-5′), 103.42 (C-6′), 23.21 (C-1″), 122.01 (C-2″), 130.09 (C-3″), 24.90 (C-4″), 24.92 (C-5″), 121.56 (C-1‴), 130.57 (C-2‴), 78.69 (C-3‴), 20.83 (C-4‴), 16.93 (C-5‴) [1]; +ve FAB-MS (*m/z*, $I_{rel.}$, %): 421 $[M+H]^+$ ($C_{25}H_{25}O_6$) (31.8), 351 (8.7), 200 (5.3), 220 (3.8) [1].

Reference

1. Ali, A.; Ali M. Isolation and structure elucidation of a new linoleiyl glycoside and flavones from the stem bark of *Morus alba* L. Future J. Pharm. Sci. 2016, 2, 82-86. https://doi.org/10.1016/j.fjps.2016.09.002
